# COVID-19 in Spain. Coming back to the “new normality” after 2 months of confinement

**DOI:** 10.1017/S1041610220001155

**Published:** 2020-10

**Authors:** Raimundo Mateos, Melchor Fernández, Manuel Franco, Manuel Sánchez

**Affiliations:** 1 Department of Psychiatry, University of Santiago de Compostela and CHUS University Hospital, Santiago de Compostela, Spain; 2 Sociedad Española de Psicogeriatría (SEPG), Barcelona, Spain; 3 GAME-IDEGA and Department of Economics, University of Santiago de Compostela, Santiago de Compostela, Spain; 4 Psychiatry and Mental Health Departments, University Rio Hortega Hospital (Valladolid) and Complejo Asistencial of Zamora, Spain; 5 Biomedicine Research Institute (IBSAL), PETRA Department, University of Salamanca, Spain; 6 Department of Geriatric Psychiatry, Sagrat Cor Hospital, Martorell, Barcelona, Spain

## The sociocultural and political background

Spain is one of the countries most affected by the coronavirus pandemic in the world, with 28,628 deaths as of today May 23, 2020. This figure, the highest after the USA, Italy and UK, underestimates the actual number of deaths attributable to the virus, since it only counts the polymerase chain reaction + cases (and Spain has not been an exception to the scarcity of these diagnostic tests). These data, like any other epidemiological data, must be interpreted in its sociodemographic, cultural and political context.

Spain is a culturally diverse country (four co-official languages, Spanish, Galician, Catalan, and Basque) and political, organized in 17 Autonomous Communities (ACs). Spain is one of the countries with the oldest population in the world: 19.3% are over 65 years old, but some ACs such as Asturias, Castilla-León, and Galicia, exceed 25%, reaching much higher percentages in the rural areas. At the same time, Spain enjoys one of the highest life expectancies in the world (83 years at birth, 21.2 at the age of 65) and one of the highest healthy life expectancy (73.8) (National Statistics Institute, 2020).

According to the official statistics, 4.3% of those over 65 years old live in some type of residential center, but coverage rates differ notably between ACs (Castilla y León 7.9%, Catalonia 4.5%, and Galicia 3.1%). In Spain, there are currently 6,240 residential centers for the elderly, which differ widely in size, ownership (public/private), profit-making, and organization. From an economic perspective, it is a rapidly growing sector (a reverse trend to that occurring in other European countries), increasing the privatization of services, increasing the size of residential centers (especially striking in Autonomies such as Galicia with an extraordinary dispersion of the population), with a high business concentration (in Galicia two groups concentrate a third of the residential places) (Fernández and Meixide, 2013).

The Spanish National Health System (“Sistema Nacional de Salud”) guarantees universal coverage and free healthcare access to all Spanish nationals, regardless of economic situation or participation in the social security network (Legido *et al*., 2020; Marczewska, 2010). Several decades ago, Catalonia created the model of care called “socio-health care.” In a simplistic definition, this model recommends that in addition to hospital care and primary care, the social health network is developed, aimed at chronic pathologies (with the majority of users over 65 years of age) (Sánchez *et al*., 2002). This model stimulated a wide debate, the term was incorporated into the technical language and to some extent has inspired healthcare reorganizations of other ACs.

At present, there is a notable difference in the cumulative incidence and mortality between the different ACs. Madrid and Catalonia accumulate more than half of the deceased (8,944 and 6,656, respectively). In relative terms, both communities remain in the group with the highest incidence and mortality rate, while, for example, Galicia is in the tail group.

The government of Spain decreed the state the alarm on March 14, which implied the strict confinement of the population. These measures have slowly begun to relax from April 26 and on May 11 the official period of “de-escalation” began, which will go at different speeds in the different regions and for the different productive sectors of the country.

## Problems faced along the past 2 months

The most widespread problem has been the shortage of personal protective equipment for health professionals and other frontline professionals (workers in residential centers, police, etc) also accompanied by a shortage of basic therapeutic materials (respirators). Trade unions and professional groups have denounced that the overload of the health system has forced health personnel to make unusual decisions and the level of work stress has become very high.

The psychiatric inpatient units were largely reconverted for coronavirus care. Resources such as electroconvulsive therapy were minimized. Outpatient mental health consultations, including those specifically for psychogeriatrics (in the few places that have them), were replaced by telephone consultations. All kinds of day centers were closed and home care was reduced.

The stress derived from confinement situations, especially severe in urban areas, had a negative effect on the behavioral disorders of people with dementia. Contributing factors were the sudden changes (in their routine, environment, and people living together) and the reduced physical activity (families could legally take the ill persons for a walk but often withdrew due to the misunderstanding of the neighborhood, who rebuked them from the balconies and sometimes even the incomprehension of the public order forces themselves).

The consequent overload of the caregivers has caused all kinds of dysfunctions, and the responses of the overwhelmed caregivers have oscillated between the extremes: At one extreme, the mechanical restraint of the patient without indication or professional supervision, inadequate hygiene and food (because the only caregiver has to be absent from work during the day), and inadequate administration of drugs (which were previously monitored in the day center). At the other extreme, hyper-adapted family members, who do not make any complaints despite the injuries caused by the patient being evident. After 2 months of confinement, the associations of relatives of people with dementia in Galicia have verified an enormous physical and cognitive deterioration of this type of patients in their home visits, even in patients who previously had only mild cognitive impairment. These associations have reported to us that their caregivers do not even report to healthcare professionals or social services about their needs, perhaps because they consider the traumatic experience they are experiencing to be “natural,” or perhaps because they do not expect any solution.

As expected, mortality has been much higher in the elderly (86% of the deceased were over 70 years old), but surely some events have created the greatest social impact:

- That at the beginning of the pandemic, there were frequent “reassuring” comments in the media that this disease “only” was going to affect the elderly.
- The mortality is disproportionately high in nursing homes. Official data have not yet been published, but reliable journalistic sources, adding official data, estimate that it exceeds 19,000, which would imply that 2/3 of all coronavirus deaths have been older people living in nursing homes (Comas-Herrera *et al*., 2020; RTVE, 2020).
- There is a consensus among families, scientific societies, and professionals in the gerontology sector that preventive measures in the nursing homes have been taken late and health care measures have been insufficient. In this regard, positions such as that of the Sociedade Galega de Xerontoloxía e Xeriatría stand out, which in a manifesto defends the effort of professionals in the nursing home sector and reminds the health system of its inexcusable duty to ensure the health of all citizens (SGXX, 2020a).
- The State Attorney General’s Office has so far opened 211 civil and 140 criminal files in this area, mainly in Madrid and Catalonia. The highest authorities have not escaped legal complaints from user associations either.
- Isolation of those who live in residences, numerous deaths produced in solitude; burials alone or with minimal family assistance. Loneliness and anguish for the elderly person, difficulty to carry out a normal grief for the relatives; great anguish of the health personnel and of the personnel who lavish care in residences, that is, debated between the impossibility of carrying out their work well and the high risk of contagion, infecting the resident and infecting their own relatives.

## Some solutions implemented or being considered

As already indicated, the reduction of face-to-face healthcare activity has been attempted to mitigate with the increase in telephone consultations and, to a lesser extent, videoconferences (telepsychiatry is still poorly developed in Spain).

- In addition, educational materials were offered for users, caregivers and professionals through web pages, either created for this purpose by the health system (eg in Galicia and Castilla León) or through the websites of scientific associations (such as the Sociedad Española de Psicogeriatría) (+Salud Mental, 2020; Sociedad Española de Psicogeriatría, 2020).
- As a result of the reorganization of care, a great reduction in demand has been observed, including emergencies, which may be due to the fear of family members to expose their elders to a source of contagion.
- In Galicia, the Associations of relatives of patients with dementia reconverted the activity of their Day Centers in Home Care. This has contributed first to identifying serious problems that were hidden from the official healthcare network and then trying to cover the needs of patients and caregivers.
- Another strategy has been the application of psychosocial interventions at home, for example, cognitive training and cognitive rehabilitation through the application of the Gradior program at a distance (Garcia-Casal *et al*., 2019).
- The serious problems that have occurred in some private residences have required that their management be assumed by the respective authorities or directly closed. Thus, in Catalonia, the nursing homes that depend on the social affairs administrations have come to do so from the Ministry of Health.
- The societies of Geriatrics and Gerontology of Spain and Galicia developed an intervention protocol in residential centers, to isolate patients diagnosed in residences in other types of establishments (e.g. hotels), and provide them with adequate medical care on site. This new model of “medicalized nursing home” was tested in Galicia and later applied in other AC (SEGG, 2020; SGXX, 2020b).
- The Democratic Union of Pensioners and Retirees of Spain and the Spanish Confederation of Senior Organizations have requested the intercession of the Ombudsman for the defense of the rights of older people in nursing homes due to the pandemic of the coronavirus.
- The Spanish Confederation of Associations of Relatives of People with Dementia (CEAFA) demands that priority to be given to the return to normality of the numerous therapeutic centers that run throughout the state to return to meet the needs of these patients and their families (CEAFA, 2020).

## Conclusions and suggestions for the future

This pandemic has not only overwhelmed health provisions for times of catastrophes, it has also been a shock to the healthcare systems and values of our society.

Regarding the first, health authorities and scientific societies agree on the need to develop telemedicine, and specifically, telepsychogeriatrics. The real test of the system of attention to mental health problems is yet to come, as some experts predict, will constitute the “fourth wave,” that of mental disorders currently contained or still being incubated (Bengoa, 2020).

Leaders and representatives of various associations in the gerontological field have published a manifesto that calls for the need to change long-term care for older people, toward a more personalized and holistic model, to which more than a thousand leaders of the sector have already adhered (Ante la crisis de COVID-19, 2020).

For decades, we have had a consensus on the principles that should guide psycho-geriatric care, and the deficiencies of psychiatric care in nursing homes are certainly known, even in countries with well-developed psychiatric services (Conn *et al*., 2007; World Health Organization, 1997).

Beyond the applause that the country’s inhabitants have dedicated to their “indispensable” professionals daily for weeks (first they were the health workers, then the tribute has been extended to other professional groups, usually “hidden”), society will have to reconsider the importance it attaches to public health and social protection systems. Let us remember what our “health heroes” have commented to the media: that what they want is not to be heroes, but to develop their work as well as possible with the appropriate resources.

Improving attention to the needs of older people requires greater knowledge and understanding of this stage of the life cycle by all of society. We will have to re-conceptualize the meaning, the roles older persons are allowed to play in our society. Only by changing our gaze toward the older people will it be possible to improve our response to future pandemics.
